# An Analysis of the Movement Trajectories of the Endangered *Acipenser gueldenstaedtii* in Ammonia-Supplemented Environments Using Image Processing Methods

**DOI:** 10.3390/ani15070900

**Published:** 2025-03-21

**Authors:** Beytullah Ahmet Balci, Güray Tonguç, Muhammed Nurullah Arslan, İlker Zeki Kurtoğlu, Tuba Sari

**Affiliations:** 1 Department of Aquaculture, Faculty of Fisheries, Akdeniz University, Antalya 07070, Türkiye; abalci@akdeniz.edu.tr; 2 Department of Management Information Systems, Faculty of Applied Sciences, Akdeniz University, Antalya 07070, Türkiye; guraytonguc@akdeniz.edu.tr (G.T.); tubasari@akdeniz.edu.tr (T.S.); 3 Department of Aquaculture, Faculty of Fisheries, Recep Tayyip Erdoğan University, Rize 53100, Türkiye; ilker.kurtoglu@erdogan.edu.tr

**Keywords:** ammonia, sturgeon, fish behaviors, image processing, object tracking

## Abstract

In this study, the effect of ammonia on the *Acipenser gueldenstaedtii* was investigated using non-invasive methods. Different concentrations (100, 200, and 400 mg·lt^−1^) of ammonium chloride (NH_4_Cl) were added to the experimental groups to simulate ammonia in aquaculture systems, and the movements of the fish were monitored, recorded, and analyzed using image processing techniques and statistical methods. For image processing operations, the optical flow Farneback object-tracking algorithm and necessary image development algorithms were implemented using Python 3.9.13 Programming language codes in the Visual Studio Code software 1.98.2 development environment. At low concentrations, it was observed that the fish made circular movements, while at high concentrations, their movements were restricted and concentrated in areas close to the water’s surface. It was observed that with the increase in ammonia concentration, the movement distances of the fish decreased, and their movements became irregular. This shows that the *Acipenser gueldenstaedtii* is sensitive to ammonia concentrations and that these concentrations affect the behavior of the fish. These findings are significant for aquaculture conditions and water quality management of the endangered *Acipenser gueldenstaedtii*, which is protected from the threat of extinction.

## Simple Summary

Sturgeons are known as an endangered species worldwide. The cultivation and monitoring of this species require large-scale operations and special conditions that need precise tracking. Traditional monitoring methods are time-consuming and costly and sometimes have limitations in terms of accuracy. Computerized image processing methods can automatically analyze and record the movement patterns, feeding habits, and social interactions of the endangered *Acipenser gueldenstaedtii*. This helps breeders better understand the health and well-being of this fish and refine their aquaculture processes. This study determined the behavioral changes exhibited when exposed to ammonia and analyzed these changes based on computer image processing techniques.

## 1. Introduction

Aquaculture is an essential sector for food security and economic development worldwide. Fish and other aquatic products are indispensable protein, fat, vitamin, and mineral sources for a healthy diet [1]. Increasing population, changing dietary habits, and decreasing natural resources have heightened the demand for aquaculture production. To meet this demand, the aquaculture sector is expanding rapidly and adopting more efficient, sustainable, and innovative methods [1,2]. Achieving these advancements with low costs and losses remains one of the most critical goals of the industry today [3].

In natural environments, ammonia can increase due to the decomposition of organic matter (e.g., fish waste or plant decay), agricultural runoff, and industrial pollution, while in aquaculture, overfeeding, inadequate water exchange, filtration system failures, and high fish stocking densities can trigger ammonia accumulation. Ammonia is very harmful to fish and shows toxic effects [4,5,6]. Generally, sturgeons require clean, well-oxygenated water and are highly sensitive to elevated ammonia levels. Ammonia is a toxic compound for fish, and its toxicity, measured as un-ionized ammonia (NH_3_), varies depending on factors such as pH, temperature, and oxygen levels. While specific ammonia tolerance ranges for the sturgeon species (e.g., *Huso huso*, *Acipenser gueldenstaedtii*, or *Acipenser ruthenus*) are not comprehensively documented in the literature, the general physiology and environmental sensitivities of sturgeons allow for some inferences. For most freshwater fish, the safe level of un-ionized ammonia is below 0.02 mg·lt^−1^; however, this threshold may be even lower for sensitive species like sturgeons. According to [7], un-ionized ammonia levels in sturgeons aquaculture should remain below 0.02 mg·lt^−1^ to ensure fish health.

The effect of ammonia on fish behavior is critical for understanding the role of water quality in fish health and welfare in aquaculture, as behavior serves as an early indicator of stress [4,6]. However, this effect remains insufficiently studied in endangered species like the *Acipenser gueldenstaedtii*. Previous studies have typically focused on physiological responses in species such as goldfish [6] or sea bass [8], with behavioral analyses remaining limited. This study aimed to address this gap using non-invasive image processing techniques.

While small amounts of ammonia can alter fish behavior, elevated concentrations disrupt essential physiological functions such as respiration, feeding, growth, and overall behavior [6,8,9,10]. Therefore, monitoring and controlling ammonia levels are vital to ensure fish welfare and optimize production. However, traditional observation methods often fall short, as they are labor-intensive, time-consuming, and limited in detecting subtle behavioral changes caused by ammonia stress [11].

There are many types of measurement, classified according to their physical properties. Today, non-destructive diagnostic systems are increasingly used in many fields, from agriculture to the aviation industry [12,13,14]. According to statistics, less than half a million fish were used for research purposes in the UK [15,16]. Computerized image processing methods can be an essential source of information for stress management, fish health, and welfare in the aquaculture industry by enabling automatic monitoring of fish behavior and rapid data analysis.

In image processing applications, operations are conducted on the digitized form of natural images from daily life using computer algorithms that have been previously developed or can be tailored to solve specific problems to obtain meaningful results. These algorithms can be applied in basic image arithmetic, point operations, geometric operations, image analysis, digital filters, feature extraction, and image transformation [17]. Image processing methods are widely utilized in the non-destructive diagnosis of fishery products, mainly fish. For instance, studies have involved species like sea bream (*Sparus aurata*) [18], sea bass (*Dicentrarchus labrax*), pointed bream (*Diplodus puntazzo*), berlam (*Merluccius merluccius*), and sea bream again (*Sparus aurata*) [19], as well as chub mackerel (*Scomber japonicus*) and blue mackerel (*Scomber australasicus*) [20], and pufferfish and goldfish [21], with [6] explicitly focusing on goldfish. Research has also examined individual or swarm movements [6], behavioral responses of zebrafish to various standard invasive procedures [22,23], fish feeding behaviors [24], fish species detection in temperate waters [25,26], and fish length measurement or estimation [19,27,28,29]. Additionally, [20] explored the automatic differentiation of fish species without human intervention, while [21] investigated distinctions between pufferfish and goldfish.

Sturgeons are a group of species with high economic value and are a commonly preferred fish in commercial aquaculture [30,31]. There are 27 species of sturgeon in the world [32], 6 of which live naturally in the Black Sea basin, namely, *Huso huso, A. gueldenstaedtii, A. nudiventris, A. ruthenus, A. stellatus,* and *A. sturio*. However, only *H. huso*, *A. gueldenstaedtii,* and *A. stellatus* species are rarely seen in the Black Sea Turkish coastal area today. Unfortunately, the other three species are reportedly extinct [33,34,35]. Sturgeons are one of the rarest species in the world, and they are valuable fish for their meat and caviar, as well as their skin and swim bladder. Their economic value is so high that it cannot be compared to other animal-based foods [36,37,38].

This study aimed to address this need by developing and testing a new method to record the motor activity of the *A. gueldenstaedtii* exposed to ammonia, utilizing computer image processing techniques to analyze behavioral changes. While ammonia stress and sturgeon conservation are relevant concerns in aquaculture, the primary focus was on creating an innovative, non-invasive tool to monitor fish motor activity under controlled ammonia exposure. By leveraging image processing, this approach seeks to overcome the limitations of traditional observation methods, offering a precise, automated system to assess stress responses. This method contributes to understanding ammonia’s impact on sturgeons and supports the broader goals of improving fish welfare and advancing sustainable aquaculture practices.

## 2. Materials and Methods

**Figure 1:**
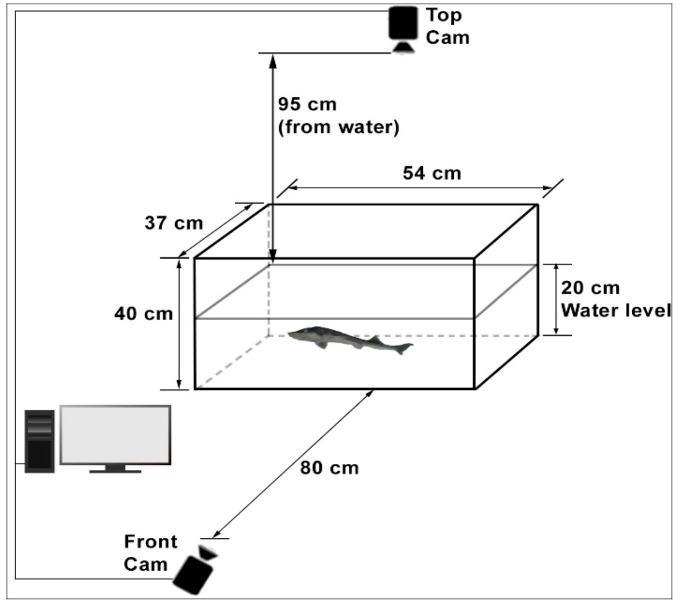
The experimental system for monitoring the *A. gueldenstaedtii* ’s behavior in glass tanks.

This study employed a non-invasive photographic method to examine fish behavior without altering their natural state. An imaging system with two cameras, positioned to view the glass tank from the front and top, was used to record movements. The setup in which the experiments were conducted is shown in Figure 1. The dimensions of the glass tank were 54 × 37 × 40 cm. This system included two high-resolution cameras, a suitable lighting scheme, and the necessary software components. The cameras mounted on the side and top of the aquariums were identical. All images were taken with these standard webcams at a 1920 × 1080 resolution, 96-dpi dot density, and 24-bit color resolution. The front camera used in the image acquisition process was located 80 cm above the water level, while the top camera was 95 cm above the water level. The cameras were placed perpendicular to the water, close enough to see only the water’s surface in the aquarium. When first installed, there were light glares on the water’s surface in the images taken from the upper camera, but these glares were eliminated by changing the location of the light source. In the side camera images, the camera’s reflection was also very faintly visible in the aquarium glass, but the image of this object was continuous. In the same place, the algorithm did not detect this, and, thus, it did not affect the work. The glass tank water height was 20 cm, which is the height at which the fish could move freely horizontally and vertically, considering the size of the fish.

This study obtained six juvenile *A. gueldenstaedtii* (approximately 1 year old) from the Recep Tayyip Erdoğan University Aquaculture Application and Research Center. One week before the experiment, the fish were acclimated in glass tanks equipped with air stones for aeration. Each tank hosted one fish to ensure isolation, and maintenance involved daily water quality monitoring. Fish lengths and weights are provided in Table 1.

Three experiments were conducted, each lasting two days, with two repetitions. On the first day, no ammonium chloride was added to the water to maintain the everyday environment. On the second day, the experimental conditions for each fish stayed the same as on the first day, except that different concentrations of ammonium chloride were added. During the study, ammonium chloride was added to the glass tanks at concentrations of 100 mg·lt^−1^ in the first setup, 200 mg·lt^−1^ in the second setup, and 400 mg·lt^−1^ in the third setup, planned according to [6] to evaluate behavioral responses to ammonia in a controlled range. The water was aerated with an air stone using a central air pump. On both days of the experiments, the movement of the fish was monitored by filming with the camera. During the study, the water temperature, pH, oxygen, ammonia, ammonium, nitrite, and nitrate content were measured at 8:55 and 19:05 in all glass tanks, chosen to align with the *A. gueldenstaedtii* ’s daily activity cycle and to capture water quality at the start and end of the daytime period [6]. The Hach-Lange HQ40d instrument (Ames, IA, USA) and Sera aqua-test kits (Heinsberg, Germany) measured these water quality criteria. After adding ammonium chloride to the glass tanks, a 10 h photography process was started for each experimental group to monitor fish movements.

**Figure 2:**
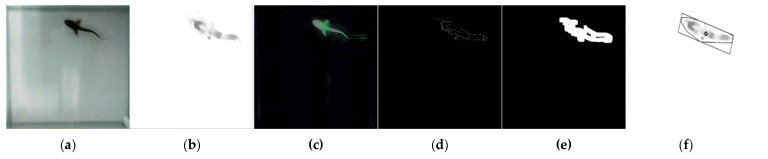
The processing stages of images taken from the glass tanks. (**a**) Original image. (**b**) Subtracted and gray-scaled image. (**c**) Optical flow Farneback result of the image. (**d**) Canny edge detected image. (**e**) Dilated image. (**f**) Polygon drawn and center calculated.

The glass tanks were photographed every second during photo shoots using cameras and Bandicam software 6.0.4.2024. By setting the value of the “Repeat screen capture” parameter in the “Image settings” section of the software to 1 s, a photo was automatically taken from the webcam once a second. In this way, the movements of the fish were monitored almost without loss. Approximately 865,000 images were obtained over two repetitions, spanning six days and 10 h per day (between 09:00 and 19:00). The images were obtained at a 1920 × 1080 resolution. The date and time information of each captured image was used as the name of that image. The obtained images were processed with the help of the OpenCV software 4.8.0.74 library and Python codes in the Visual Studio Code software development environment in line with the algorithm developed for this study. In the study, an attempt was made to follow the fish in the software using images of a single fish. In the images taken from the front camera, the long side was designated as X and the short side as Y; in the images taken from the top camera, the long side was designated as X and the short side as Z. The X coordinate information of the fish detected in the images taken by both the front and top cameras was simultaneously evaluated by matching. For matching, fish coordinates were determined in the top and side images. The data in the images taken on the same date and time were combined in the same row in the dataset, and after this stage, work began on the three-dimensional location information of the fish. As a result of processing each image, the date–time of the image, the X and Y coordinates of the fish (in pixels), the area covered by the fish in the image, and the angle of the fish were obtained. In addition, using these data, the time elapsed since the start of the experiment, the X and Y (and Z) coordinates of the fish in millimeters, and the distance swam by the fish between the two images were determined. In the developed algorithm, each image was opened individually (Figure 2a). The opened image was cropped according to the habitat dimensions of the fish determined by the researchers to shorten the processing time and prevent the acquisition of erroneous information. The reference image taken while the glass tank was empty before the study started was subtracted from each image. The subtracted image was converted to grayscale (Figure 2b) and subjected to the optical flow Farneback process, an object-tracking algorithm, to track the fish (Figure 2c). In the optical flow Farneback process [39,40], a comparison was made between the earlier image and the examined image, and the moving object’s position and its motion information were extracted from the photos. The canny edge detection algorithm (Figure 2d) and dilate process (Figure 2e) were applied to detect the edges of the fish whose motion information was extracted from the image created with this information. At this stage, the area occupied by the fish in the image appeared in black and white. Finally, a polygon was drawn around this area, covering this area as a minimum, and its center was calculated (Figure 2f). This point represents the coordinates of the fish’s location.

The fish position and movement data obtained in this study were also examined statistically. Since it would not be appropriate to analyze the data taken from the fish every second for this process, 10 s averages of the speed values were calculated, and 3600 speed information points were statistically processed for each experiment. A standard distribution test of the measured values was performed using the single-sample Kolmogorov–Smirnov test [41]. The differences between the groups were evaluated using the Kruskal–Wallis statistical test, which is a non-parametric test [42]. Since a significant difference was found between the groups using the Kruskal–Wallis test, a post hoc test was applied to show which groups differed [43]. The Dunn test, a type of post hoc test, was evaluated in this study using Bonferroni correction. Statistics were evaluated at a confidence interval of 95% (*p* < 0.05). All statistical analyses were performed on the Colab platform using Python 3.9.13 and libraries containing statistical functions [44].

## 3. Results

Table 2 shows the water criterion values of the control and experimental group glass tanks measured in the experiments. Measurements of water parameters were conducted in the morning and evening hours (08:55 and 19:05).

**Figure 3:**
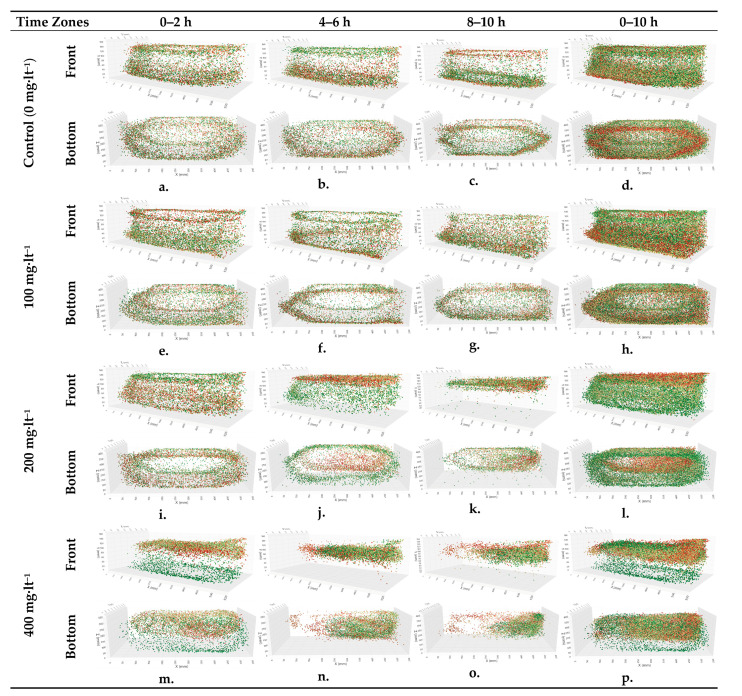
The three-dimensional positions of the *A. gueldenstaedtii* in the tank, as determined by the cameras during the experiment (front and bottom camera views).

The graphs in Figure 3 show the coordinates of the *A. gueldenstaedtii* at the 0–2, 4–6, 8–10, and 0–10 h intervals from the beginning of the study. Intensity graphs showing the positions of the fish in the glass tanks between the hours are given. The graphs are provided at two-hour intervals to observe the changes better. The data obtained from the image processing steps mentioned above were transferred to Microsoft PowerBI software 2.140.1577.0. This tool used the fish’s X, Y, and Z coordinates for each second to obtain the images below. The X, Y, and Z axes in the graph represent the width, height, and depth values of the glass tank, respectively. The graph of a single control group, selected from multiple control groups that were observed to show no significant differences from one another in the experimental scenario, is provided.

In this study, one control and three experimental groups—100, 200, and 400 mg·lt^−1^ ammonium chloride—were added to the experimental groups, respectively. The graphs in the first three columns in Figure 3 show the coordinates of the fish in the glass tanks at the 0–2, 4–6, and 8–10 h intervals from the start of the experiment from a three-dimensional perspective. The work started at 09:00 every morning and ended at 19:00. The 0–2 h interval corresponded to the morning hours; when the working hours began, the 4–6 h interval corresponded to the midday hours, and the 8–10 h interval corresponded to the end-of-day hours. The graphs in the last column show the fish coordinates during the entire period of the relevant day. The colors of the points on the graph change from green to red according to the time the data representing the fact were obtained. In other words, green dots show early times, and red dots indicate later times. Each graphic above was examined in detail from different angles from a three-dimensional perspective in the software in which the graphics were drawn, and the following evaluations were made:

In the control group (Figure 3a–c), it was determined that the *A. gueldenstaedtii* wandered around the whole area of the glass tank in the morning hours by making more circular movements (Figure 3a), spending approximately 60% of the time in circular patterns across the tank’s central and peripheral zones.

*A. gueldenstaedtii* also roamed the whole glass tank in the afternoon hours, while in the later hours, it mostly wandered around the bottom of the glass tank. This is illustrated with red dots. In the following minutes, the fish swam more toward the bottom and were thought to be searching for food (Figure 1b). In the graph representing the next step, the movements of the fish continued to occur at the bottom and then shifted closer to the water’s surface. This situation was interpreted as a movement to the surface to balance the air (Figure 3c). It is generally thought that fish come to the surface at certain times to balance the air in the swim bladder [6]. When the coordinates of the fish in the control group’s glass tank were examined over the 10 h period from morning to evening, it was seen that the fish circulated homogeneously throughout the glass tank (Figure 3d).

In the case of adding 100 mg·lt^−1^ of ammonium chloride in the experimental groups, it was observed that the fish made a homogeneous wandering movement first in the bottom parts and then in the upper parts in the morning hours (Figure 3e). At noon, the effects of ammonium chloride started to be seen, in that fish movements were circular on the surface in the first part and slightly concentrated in the lower bottom corners in the following process (Figure 3f). During the final hour of the experiment, the fish displayed consistent circular movements along the bottom edges, as indicated by the concentrated trajectories in Figure 3g.

When the 100 mg·lt^−1^ experiment was evaluated for the full 10 h period, it was seen that the fish roamed in a continuous and circular motion near the bottom in the later hours. The fact that this movement, which took place homogeneously in the control group, was contrary to what was expected in the experimental group was interpreted as the fish being under stress (Figure 3h).

In the 200 mg·lt^−1^ experimental group, it was observed that the fish were not affected much by the chemical in the morning hours, which is when the ammonium chloride was applied. They showed circular wandering behavior in the upper parts of the glass tank early in the morning and in the bottom parts later in the morning (Figure 3i). At noon, their density was higher near the upper surface, and these movements appeared circular when viewed from below (Figure 3j). It was observed that the fish moved entirely on the water’s surface in the evening hours, with movements at the bottom being almost absent; meanwhile, in the following hours, they made short circular movements in a particular area of the glass tank (Figure 3k). When all the data of the 200 mg·lt^−1^ experimental group provided in Figure 3l were evaluated, it was determined that the smooth circular movements throughout the glass tank in the morning hours, as mentioned above, were disrupted toward the end of the day and occurred in areas close to the water’s surface. In the experimental group treated with 400 mg·lt^−1^ of ammonium chloride, it was observed that while the fish were mainly in the bottom areas in the early morning, they were mostly near the water’s surface in the later hours, and there were partial decreases in their rhythmic circular movements (Figure 3m). The deterioration in movements increased more at noon, leading to irregular swimming movements characterized by inconsistent paths and abrupt changes in direction compared to the control group, occurring very close to the water’s surface and in more restricted areas (Figure 3n).

The deterioration in movement observed at noon continued to intensify, and the fish were concentrated in a smaller area of a specific part of the glass tank. Condensation can be seen more clearly in the bottom view of the three-dimensional graph (Figure 3o). There was an air tube in the rear-left corner of the glass tank, and the experimental group tended to move away from the air stone in the mentioned time period. When all the coordinates of the fish in the 400 mg·lt^−1^ experiment were examined (Figure 3p), the findings showed irregular and dense distributions. In the early hours of the day, they were sparsely located at the bottom, while later in the day, especially in the final hours, they were found in a small, dense area close to the water’s surface, in the upper-right part of the glass tank.

**Figure 4:**
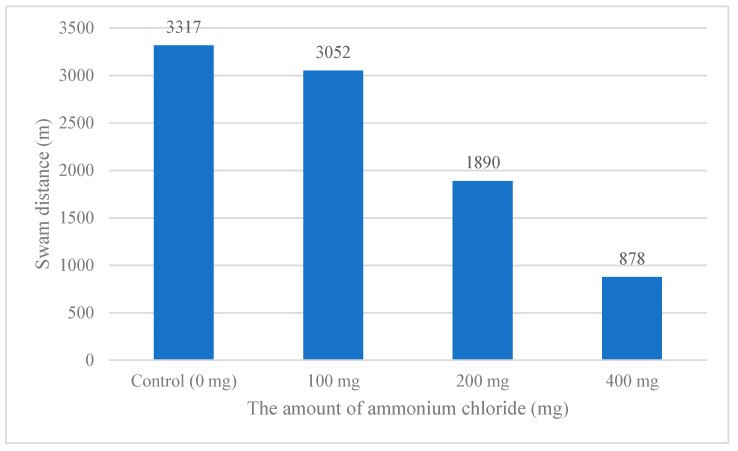
Total swimming distances of *A. gueldenstaedtii* in experimental groups during the study.

Looking at the distances the *A. gueldenstaedtii* swam in the glass tanks, they averaged 3317, 3052, 1890, and 878 m across the two replicates for each ammonia concentration, respectively. The amount of ammonium and the *A. gueldenstaedtii* displacement varied inversely (Figure 4).

The fish location and movement data obtained in the study were subjected to statistical analysis. Since using the data which recorded every second of the fish directly during the analysis process was not deemed appropriate, the 10 s averages of the speed values were calculated. In this way, 3600 speed data points were included in the statistical analysis for each trial (Table 3). Descriptive statistics of the speeds for the control group and different dosages of ammonia are provided. While the control group stands out as the group with the highest average speed, its standard deviation is also higher than that of the other groups. As the amount of ammonia increased, the standard deviation values, which represent the difference between the highest and lowest speeds of the fish, and the average speed decreased.

Table 4 shows a statistically significant difference between the medians of the groups in terms of speed, based on the *p*-values obtained from the post hoc analysis. There was no statistically significant difference between the control group and the 100 mg·lt^−1^ experimental group regarding fish speed, but there was a difference between the control group and all other groups.

## 4. Discussion

In the setup, the level of the water was determined by considering the size of the fish, which was determined to be 20 cm, to allow the fish to perform movements efficiently. In addition, efforts were made to keep the water level as low as possible to reduce the size of the image to be processed so that the processing time did not extend during image processing. The water level was set as 5 cm for fish with a body length of 6 cm and a body height of 2 cm [6]. In the study, an image was taken every second, which was sufficient to follow the movements of the fish fluently. This process was reported by [18] at three frames per second, Ref. [45] at 29 frames per second, and by [23] at 30 frames per second. During image acquisition, some reflections partially affected the processing of the images, and efforts were made to prevent them using software [6]. Additionally, a black cloth layer was placed at the bottom of the glass tank to avoid reflection.

In [46], the authors stated that in simple problems, it would be more appropriate to use algorithms with less processing time and computational complexity, which will be required during training and working. In this study, a single fish was used, and unlike profound learning-based studies that determine the fish and its species, only a tracking algorithm that tracks the movements in the acquired image was used. Thus, laborious processes such as dataset preparation and training for deep learning models were avoided. Studies using one fish, more than one fish, and more than one fish species can be found in the literature [23,24,47]. In addition to basic image processing algorithms [18,20,21,23,48], the researchers also used algorithms they developed in their studies [28,47]. In this study, in which one sturgeon was followed per glass tank, the optical flow Farneback algorithm was employed to track the fish, as well as algorithms based on basic image processing methods to process the image better before and after the tracking process, thus providing more accurate results. Most of these algorithms are provided in the OpenCV image processing library.

In [6], the researchers applied three ammonia concentrations (100, 200, and 400 mg·lt^−1^) to goldfish, observing behavioral trajectories that showed reduced movement, like our findings with the *A. gueldenstaedtii*. They also tested a 600 mg·lt^−1^ dose, which proved lethal after nine hours, causing fish mortality. Likewise, in this study, we applied the same three concentrations (100, 200, and 400 mg·lt^−1^) to the *A. gueldenstaedtii* to assess motor activity changes, opting not to use 600 mg·lt^−1^ due to its potentially lethal impact.

In [24], in the resting state, the fish moved around the tank without a specific movement pattern. The researchers noted that after performing one of the procedures, the fish tended to spend most of their time at the bottom of the tank rather than occupying the entire tank, and they concentrated on localized areas within the tank where they were generally less active. This study determined that in all control groups, the *A. gueldenstaedtii* made more circular movements across the entire region of the glass tank, and circulated homogeneously in the glass tank in the coordinate graph. When the coordinates of the control groups were examined, no significant difference was observed between any control groups, which was also confirmed statistically (Table 4). In the case of adding 100 and 200 mg·lt^−1^ of ammonium chloride to the experimental groups, in the morning hours, the fish made homogeneous movements around the bottom of the glass tank and then the upper parts, and the effect of ammonium chloride increased after six to eight hours. The movements on the bottom were replaced by circular movements on the surface as time passed. It was determined that the fish started to form small densities in the corners. In the last hour after the addition of 200 mg·lt^−1^ of ammonium chloride, the movements of the fish generally increased in circular motions along the bottom edges. The same situation was interpreted as the result of the stress and negative effect of ammonium chloride on the fish in the last hour of the 400 mg·lt^−1^ experimental group, as the short circular movements on the water’s surface and in a particular area of the glass tank intensified. It was thought that the fish roamed in the bottom parts of the glass tank more in the following hours due to their anatomical condition and search for food. It is believed that the fish came to the surface at certain times to balance the air in the swim bladder. In [6], the authors reported that a significant decrease in the movements of the fish occurred in environments with ammonia compared to the everyday environment and that the fish were immobile in the water. In our study, the observation of irregular movements initially detected in the right-rear part of the glass tank in the *A. gueldenstaedtii*, followed by a marked intensification of these movements, is consistent with the findings of [6,24]. These patterns suggest that ammonia exposure triggers distinct behavioral responses in sturgeon, aligning with prior research documenting similar changes under stress conditions. This consistency reinforces the reliability of our observations and supports the application of computer image processing to quantify such behaviors accurately. This consistency stems from the distance swam by the fish in the glass tanks (0 mg·lt^−1^ (control), 100 mg·lt^−1^, 200 mg·lt^−1^, 400 mg·lt^−1^; 3320, 3050, 1890, and 880 m, respectively), decreasing as the ammonia concentration increased, supporting this reduction. The shortest distance was observed in the 400 mg·lt^−1^ experimental group. These findings show the negative effect of ammonium chloride on sturgeons. In [6], the researchers reported that there may be problems in the aquaculture environment when the fish are not active, according to the behavioral trajectory. These findings demonstrate the negative impact of ammonium chloride on sturgeon fish. According to [6], reduced fish activity, as observed through behavioral trajectories, may indicate underlying issues in the aquaculture environment, such as ammonia-related stress.

This study extends beyond our controlled conditions, providing potential insights into the behavior of the *A. gueldenstaedtii* in both natural and aquaculture systems. By capturing motor activity responses to ammonia, our image processing method can be adapted to study fish in natural environments affected by pollutants or aquaculture techniques for water quality control, and the monitoring of fish behavior under various scenarios can be improved. In practice, our findings of reduced locomotion and aggregation behavior at higher ammonia levels could inform strategies to mitigate ammonia stress in aquaculture by improving water quality, such as triggering timely water treatment or aeration when these behavioral indicators occur. Furthermore, while ammonia drove the responses observed here, similar reductions or changes in activity could also be caused by other environmental stressors, such as low oxygen or extreme temperatures, suggesting that this approach could be extended to explore a broader range of effects on sturgeon behavior.

## 5. Conclusions

The effect of different ammonia concentrations on the behavior of juvenile *A. gueldenstaedtii* individuals was investigated using a computerized image processing approach. The three-dimensional trajectory of the fish was drawn, and the behavior of the fish was analyzed. The findings obtained using this method showed that the *A. gueldenstaedtii* juveniles were sensitive to ammonia and had a significant decrease in swimming movements. With this proposed approach, it is possible to study the behavior of fish, changes in the aquaculture environment, diseases, and more, using a simple, effective, and non-invasive method. By applying image analysis methods across all areas of the aquaculture industry, productivity can be increased, sustainable development can be promoted, quality can be improved, and losses can be minimized. It is already evident that, in the future, the aquaculture sector will benefit from greater interest and contributions through wireless sensor networks, image processing technology, and artificial intelligence algorithms. Since the water quality parameters in fish habitats have deteriorated because of climate change and environmental pollution, it is thought that fish in their natural environment are also exposed to the adverse effects seen in this study. In future studies, studies with deeper water levels and higher vertical distribution sensitivity would be more interesting. Conducting similar studies with different stimulants and on fish of varying sizes will help uncover the potential adverse effects on fish behavior under changing environmental conditions.

## Figures and Tables

**Table 1:** The length and weight of the *A. gueldenstaedtii* fish used in the experiments.

	Experiment 1 (100 mg·lt^−1^)		Experiment 2 (200 mg·lt^−1^)		Experiment 3 (400 mg·lt^−1^)	
	Glass Tank 1	Glass Tank 2	Glass Tank 1	Glass Tank 2	Glass Tank 1	Glass Tank 2
**Length** (**cm**)	16.5	16.6	19.8	19.1	19.8	19.9
**Weight** (**g**)	14.2	12.1	18.3	16.5	19.5	18.8

**Table 2:** Water parameters measured during the experiments on the *A. gueldenstaedtii*.

Water Parameters				pH	Temperature (°C)	O2 (mg·lt^−1^)	NO2 (mg·lt^−1^)	NO3 (mg·lt^−1^)	NH3 (mg·lt^−1^)	NH4 (mg·lt^−1^)
**Experiment 1** (**100 mg·lt^−1^**)	**Glass Tank 1**	**Day 1**	**Morning**	7.5	19.1	8.76	0	0	0.5	0.5
			**Evening**	8	19.3	8.74	0	0	0.5	0.5
		**Day 2**	**Morning**	7.5	19.2	8.73	0	0	>10	>10
			**Evening**	8	19.1	8.61	0	0	>10	>10
	**Glass Tank 2**	**Day 1**	**Morning**	7.5	19.3	8.78	0	0	0.5	0.5
			**Evening**	8	19.1	8.77	0	0	0.5	0.5
		**Day 2**	**Morning**	7.5	19	8.75	0	0	>10	>10
			**Evening**	8	19.1	8.6	0	0	>10	>10
**Experiment 2** (**200 mg·lt^−1^**)	**Glass Tank 1**	**Day 1**	**Morning**	7.5	18.9	8.2	0	0	0.5	0.5
			**Evening**	8	18.8	8.15	0	0	0.5	0.5
		**Day 2**	**Morning**	7.5	18.9	8.3	0	0	>10	>10
			**Evening**	8	18.9	8.1	0	0	>10	>10
	**Glass Tank 2**	**Day 1**	**Morning**	7.5	18.8	8.17	0	0	0.5	0.5
			**Evening**	8	18.9	8.05	0	0	0.5	0.5
		**Day 2**	**Morning**	7.5	18.8	8.27	0	0	>10	>10
			**Evening**	8	19	8.22	0	0	>10	>10
**Experiment 3** (**400 mg·lt^−1^**)	**Glass Tank 1**	**Day 1**	**Morning**	7.5	18.7	8.08	0	0	0.5	0.5
			**Evening**	8	18.9	8.01	0	0	0.5	0.5
		**Day 2**	**Morning**	7.5	18.7	8.03	0	0	>10	>10
			**Evening**	8	18.9	8.01	0	0	>10	>10
	**Glass Tank 2**	**Day 1**	**Morning**	7.5	18.6	8.28	0	0	0.5	0.5
			**Evening**	8	18.8	8.06	0	0	0.5	0.5
		**Day 2**	**Morning**	7.5	18.8	8.3	0	0	>10	>10
			**Evening**	8	19	8.15	0	0	>10	>10

**Table 3:** Speed values between the experimental *A. gueldenstaedtii* groups.

Descriptive Statistics	Control	100 mg·lt^−1^	200 mg·lt^−1^	400 mg·lt^−1^
**Mean**	91.24	83.87	52.11	24.22
**Standard Deviation**	39.90	23.88	22.95	9.34
**Minimum**	0.00	0.00	0.00	0.00
**Maximum**	189.20	148.18	159.11	122.63

**Table 4:** Post hoc test results of the fish movement speed values (Dunn test results, *p*-values).

	Control Group	100 mg·lt^−1^ Group	200 mg·lt^−1^ Group	400 mg·lt^−1^ Group
**Control Group**	X *p* = 1.000000	No difference *p* = 0.087292	Difference *p* = 0.000000 × 10^0^	Difference *p* = 0.000000 × 10^0^
**100 mg·lt^−1^** **Group**	No difference *p* = 0.087292	X *p* = 1.000000	Difference *p* = 1.472522 × 10^−291^	Difference *p* = 0.000000 × 10^0^
**200 mg·lt^−1^** **Group**	Difference *p* = 0.000000	Difference *p* = 1.472522 × 10^−291^	X *p* = 1.000000	Difference *p* = 3.306043 × 10^−305^
**400 mg·lt^−1^** **Group**	Difference *p* = 0.000000	Difference *p* = 0.000000	Difference *p* = 3.306043 × 10^−305^	X *p* = 1.000000

## Data Availability

Data are available upon request.
